# “Who are you calling rural?”: An Analysis of Classification Codes Evaluating Disadvantage in Rural Regions and their Correlation in an Appalachian Kentucky Cohort

**DOI:** 10.1002/alz70860_106278

**Published:** 2025-12-23

**Authors:** Lauren Bojarski, Cassity High, Christopher J McLouth, Erin L. Abner, Rada Choate, Elif Pinar Coskun, Elizabeth K. Rhodus, Lauren Whitehurst, Frederick A Schmitt, Linda Jo Van Eldik, Gregory Jicha

**Affiliations:** ^1^ University of Kentucky College of Medicine Department of Neurology, Lexington, KY, USA; ^2^ University of Kentucky College of Medicine, Lexington, KY, USA; ^3^ University of Kentucky College of Medicine Department of Biostatistics, Lexington, KY, USA; ^4^ University of Kentucky College of Medicine Department of Epidemiology, Lexington, KY, USA; ^5^ Department of Epidemiology and Environmental Health, University of Kentucky, Lexington, KY, USA; ^6^ University of Kentucky College of Medicine Sanders‐Brown Center on Aging, Lexington, KY, USA; ^7^ University of Kentucky College of Public Health, Lexington, KY, USA; ^8^ University of kentucky, Department of Neurology, Lexington, KY, USA; ^9^ University of Kentucky, SBCoA, Lexington, KY, USA; ^10^ University of Kentucky College of Medicine Department of Behavioral Science, Lexington, KY, USA; ^11^ University of Kentucky College of Arts and Sciences Department of Psychology, Lexington, KY, USA; ^12^ University of Kentucky College of Medicine Department of Neurosciences, Lexington, KY, USA; ^13^ University of Kentucky College of Medicine Department of Neurolog, Lexington, KY, USA

## Abstract

**Background:**

Older adults living in “rural” regions have an increased risk for developing impaired cognition (both mild cognitive impairment and dementia) and, while “rural” classification is a recognized indicator of under‐represented group (URG) status by NIH definitions, there is no standard definition of rurality designated for use in research. There are multiple measures of disadvantage that may correspond to “rural” designation, which is evaluated in this analysis.

**Method:**

Using a cross‐sectional analytic approach, 790 active University of Kentucky Alzheimer's Disease Research Center Cohort participants were identified for inclusion based on presence of demographic characteristics. Health Resources and Services Administration (HRSA) Rural, Medically Underserved Areas/Health Professional Shortage Areas (MUA/HPSA), Area Deprivation Index (ADI) State Decile and National Percentile scores, and Rural‐Urban Continuum Codes (RUCC) were identified for each participant based on their address data; proportions of the cohort meeting study definitions were assessed, as was concordance between each classification. For ADI State Decile, a cutoff set at ≥ 7th decile, for ADI National Percentile a cutoff set at ≥70%, and for RUCC a cutoff set at ≥4 (i.e., all non‐metro categories).

**Result:**

Proportions defined as living in a rural or disadvantaged area varied by classification, ranging from 13% to 27% of the cohort. Less than 1% of the total cohort met all 5 classifications, suggesting a significant lack of overlap in these constructs. HRSA Rural and MUA/HPSA classifications overlapped in 19% of the cohort (*p* <0.0001). These HRSA classifications were highly associated with ADI State decile and National percentile scores (*p* <0.0001), but not with RUCC codes (*p* = 0.05). ADI State decile and National percentile codes were highly associated with one another (*p* <0.0001), but not with RUCC codes (*p* = 0.48 & 0.59 respectively).

**Conclusion:**

These data demonstrate significant overlap in HRSA and ADI classification systems, but not between these classifications and RUCC codes. We propose that the meaning of rurality remains broad and defined by multiple aspects, and care should be taken to not conflate these different classification systems as each may serve a unique purpose, several may be related to each other, but none are fully interchangeable.

